# Impact of personality status on the outcomes and cost of cognitive–behavioural therapy for health anxiety

**DOI:** 10.1192/bjp.bp.115.173526

**Published:** 2016-09

**Authors:** Rahil Sanatinia, Duolao Wang, Peter Tyrer, Helen Tyrer, Mike Crawford, Sylvia Cooper, Gemma Loebenberg, Barbara Barrett

**Affiliations:** **Rahil Sanatinia**, MD, Centre for Mental Health, Imperial College, London; **Duolao Wang**, PhD, Department of Clinical Sciences, Liverpool School of Tropical Medicine, Liverpool; **Peter Tyrer**, FMedSci, **Helen Tyrer**, MRCGP, PhD, **Mike Crawford**, FRCPsych, **Sylvia Cooper**, BSc, Centre for Mental Health, Imperial College, London; **Gemma Loebenberg**, MSc, North West London Clinical Research Network, Hammersmith Hospital, London; **Barbara Barrett**, PhD, King's Health Economics, King's College London, London, UK

## Abstract

**Background**

Health anxiety, hypochondriasis and personality disturbance commonly coexist. The impact of personality status was assessed in a secondary analysis of a randomised controlled trial (RCT).

**Aims**

To test the impact of personality status using ICD-11 criteria on the clinical and cost outcomes of treatment with cognitive–behavioural therapy for health anxiety (CBT-HA) and standard care over 2 years.

**Method**

Personality dysfunction was assessed at baseline in 444 patients before randomisation and independent assessment of costs and outcomes made on four occasions over 2 years.

**Results**

In total, 381 patients (86%) had some personality dysfunction with 184 (41%) satisfying the ICD criteria for personality disorder. Those with no personality dysfunction showed no treatment differences (*P* = 0.90) and worse social function with CBT-HA compared with standard care (*P*<0.03) whereas all other personality groups showed greater improvement with CBT-HA maintained over 2 years (*P*<0.001). Less benefit was shown in those with more severe personality disorder (*P*<0.05). Costs were less with CBT-HA except for non-significant greater differences in those with moderate or severe personality disorder.

**Conclusions**

The results contradict the hypothesis that personality disorder impairs response to CBT in health anxiety in both the short and medium term.

It has been established that both medical and psychiatric out-patients have a high prevalence of hypochondriasis and its related new diagnosis, health anxiety.^1,2^ Patients attending out-patient clinics also have a high prevalence of personality disorder, usually between 25 and 35%, and in some cases these personality characteristics could in themselves be regarded as hypochondriacal.^1^ In addition, there is robust evidence that the presence of personality disorder impairs response to treatment and worsens outcome in depression^3,4^ and to a lesser extent in anxiety and obsessive–compulsive disorders.^5–8^ Personality disorder has also been found to be a predictor of higher costs in the long term among people with depression.^9^ To date, there are no published studies examining the effect of personality disorder on the outcome of health anxiety or on the response to treatment. In the presence of personality disorder, the benefits of cognitive–behavioural therapy (CBT) for anxiety and depression may not be as marked as those from antidepressant drug treatment.^10,11^ Hypochondriacal personality features may also impair the outcome of common mental disorders in the long term.^12^ The status of health anxiety in current psychiatric classifications has also been a subject of debate, particularly as the diagnosis of hypochondriasis has been removed from DSM-5,^13–15^ and partly replaced by ‘illness anxiety’.^16^ In ICD-11 health anxiety may or may not be a formal diagnosis in its own right, and the diagnosis of hypochondriasis (together with illness anxiety) could still be retained. Because of this uncertainty we used a two-stage process of recruitment to a planned randomised study of psychological treatment to ensure that we recruited those who not only had hypochondriasis but also significant associated anxiety, so that those who were primarily depressed and hypochondriacal were not included. At the time the trial began there was only limited evidence of the efficacy of psychological treatments for this condition.^17^ In addition, because of the strong associations between personality disorder and anxiety, confirmed recently in another study,^18^ the assessment of personality status was included in the planned protocol. This was a randomised controlled trial (RCT) of the cost-effectiveness of a modified form of cognitive–behavioural therapy for health anxiety (CBT-HA).^19^ Two hypotheses related to personality were given in the published protocol.^19^ First, that CBT-HA would be less effective in patients who had additional personality disorder and second that such comorbid personality disorder would be associated with increased costs over a period of 2 years.

## Method

### Study design

The Cognitive behaviour therapy for Health Anxiety in Medical Patients (CHAMP) trial was a pragmatic RCT (ISRCTN14565822); full details of the trial are given elsewhere.^19^ In brief, the study recruited patients attending medical out-patient clinics and randomised them to either 5–10 sessions of CBT-HA (from initially naive but subsequently trained therapists) or to standard care in primary and secondary care clinics. Patients attending cardiology, endocrine, gastroenterology, neurology and respiratory medicine clinics were included from six hospitals in London, Middlesex and North Nottinghamshire. The recruitment process was as follows: patients, with their consent, who were attending clinics completed the short form of the Health Anxiety Inventory (HAI),^20^ a self-rated scale of 14 questions with a score range of 0–42. Those that scored 20 or more on the scale (a point that has previously been shown to discriminate between those who have persistent worries over health and those who show normal variation^20,21^) were invited to take part in the trial and an information sheet about the study was given. In addition, the initial assessment involved asking key questions from the Structured Clinical Interview for DSM-IV^22^ covering the formal diagnosis of hypochondriasis. The inclusion criteria were patients aged between 16 and 75 years, living in the area covered by the hospital, with sufficient understanding of English to read and complete study questionnaires and interviews, and who had given written consent for interviews, audiotaping of 50% of treatment sessions, and for access to their medical records.^19^ All those who satisfied the inclusion criteria and hypochondriasis diagnosis were then offered randomisation to the trial, and, if they agreed, full baseline assessments were completed and written informed consent obtained. This ensured a population who had anxious hypochondriasis primarily. Follow-up data were collected at 3, 6, 12 and 24 months. The study was approved by the North Nottingham Ethics Committee (08/H0403/56) before the start of data collection.

### Assessments

The primary outcome measure was the HAI score change from baseline after 1 year.^20^ Other measures included generalised anxiety and depression using the Hospital Anxiety and Depression Scale (HADS-A and HADS-D),^23^ health-related quality of life using the short Euroqol measure (EQ-5D),^24^ and social functioning using the Social Functioning Questionnaire (SFQ).^25^ All measures were recorded at baseline, 6, 12 and 24 months (with the exception of the HAI which was also recorded at 3 months). Assessments were made completely independently by research assistants. Service-use data for the economic evaluation were collected at baseline, 6-, 12- and 24-month follow-up using the Adult Service Use Schedule (AD-SUS), a self-report instrument assessed in interview and designed on the basis of previous economic evaluations in adult mental health populations.^26^

Personality assessment was carried out using the quick version of the Personality Assessment Schedule (PAS-Q),^27^ which records both the severity and the type of personality disorder using a four-point scale (see online supplement DS1). This contains a series of screening questions for each area of personality dysfunction, and those that score positive are asked further questions. The PAS-Q was administered by a trained research assistant, and the assessment forms include both numerical ratings and written comments on each of the sections. During the course of the study the Working Group for the Reclassification of Personality Disorder in ICD-11 completed its initial work on a new system of classification based on severity criteria (April 2010).^28^ The ICD-11 classification at that stage is summarised (Appendix). Subsequently, R.S., P.T. and G.L. reclassified the personality status of the patients in the study to convert them to ICD-11 severity equivalents by examining the PAS-Q data and written comments^29^ as well as interviewing assessors if the data were not clear. For 30 of the assessments R.S. and P.T. completed independent assessments and achieved a good level of agreement (kappa (κ) = 0.84, 95% CI 0.60–1.0). The ICD-11 classification will be published in 2017.

### Randomisation and masking

Randomisation to the two treatment groups was carried out by an independently operated computerised system (Open-CDMS), with a computer-generated random sequence using block randomisation with varying block sizes of four and six. The allocation sequence was not available to any member of the research team until databases had been completed and locked.

### Statistical analysis

The calculation of the sample size for the main study has been described previously;^19^ it was powered to assess the superiority of CBT-HA over standard care after 1 year, with the HAI score as the main outcome measure. The current study was a secondary analysis of the outcomes for different levels of severity of personality disturbance and so no formal sample size calculation was performed. The primary end-point (HAI) was analysed using a mixed model with time, treatment group and time × treatment interaction as fixed effects, baseline measurement as covariate and patient as random effect by personality severity group in order to test for the first hypothesis, that the CBT-HA would be less effective in participants with a personality disorder. The treatment differences between the four ICD-11 personality groups were calculated at each time point (3 months for HAI only), 6 months, 1 year and 2 years). Other secondary end-points were analysed in the same way. All analyses were based on the intention-to-treat principle. At baseline, continuous data were expressed as means and standard deviations and compared using analysis of variance (ANOVA). Categorical data were summarised using number (%) and compared using chi-squared tests.

### Economic analysis

The planned economic evaluation is described in detail elsewhere.^19^ Total costs were calculated by combining the service-use data collected from the AD-SUS together with hospital use from electronic records with nationally applicable unit costs.^30–32^ Costs were calculated and analysed in UK pound sterling for the financial year 2008–2009 and were discounted in the second year at a rate of 3.5% as recommended by the National Institute for Health and Care Excellence.^33^ Complete case analysis was used for the economic evaluation.^19^ The second hypothesis, that participants with personality disorder would have increased costs was explored through the examination of differences in costs over the 24-month follow-up period between ICD-11 groups. Analysis was performed using ordinary-least-squares regression as is appropriate for cost data, with the robustness of the tests confirmed using bias-corrected, non-parametric bootstrapping.^34,35^ Differences in all analyses were adjusted for baseline costs and randomised group. We tested for differences between complete cases and missing cases using key baseline characteristics and found no significant difference between groups – only the complete cases appear in the analysis.

## Results

In total, 445 patients were randomised in the study but one patient was referred and randomised twice – both times to the standard care group – and the first date was taken for inclusion. All 444 patients had their personality status assessed at baseline (Appendix). Nine patients died during the study, six in the standard care group, three in the CBT-HA group. Of the patients who died one had no personality dysfunction, four had personality difficulty, one had mild personality disorder and three had moderate personality disorder.

Using the ICD-11 classification only 63 (14.2%) had no personality dysfunction but 197 (44.3%) had personality difficulty (a subthreshold condition not qualifying for disorder). Only three people assessed had severe personality disorder and so they were included with the moderate group as their numbers were too low for analysis. No differences in patient characteristics at baseline were identified and there was an even spread of men and women and a similar age profile between the ICD-11 personality groups (Table 1). However, there were significant differences in symptoms of health anxiety and generalised anxiety, depression and social functioning at baseline; participants with moderate to severe personality disorder had significantly higher scores than those with no personality disturbance (Table 1). There were no differences in total cost at baseline.

**Table 1:** Patient characteristics, clinical ratings and cost at baseline by ICD-11 personality level^a^

	Personality level				
	0 (*n* = 63)	1 (*n* = 197)	2 (*n* = 142)	3–4 (*n* = 42)	Statistics^b^ *P*
Gender, *n* (%)					
Female	29 (46.0)	109 (55.3)	76 (53.5)	22 (52.4)	0.642
Male	34 (54.0)	88 (44.7)	66 (46.5)	20 (47.6)	

Age, years					
Mean (s.d.)	48.6 (14.8)	49.5 (13.6)	47.5 (13.6)	47.9 (11.3)	0.592
Minimum–Maximum	18.3–73.9	17.3–74.3	17.0–75.5	21.7–72.4	

Health Anxiety Inventory, mean (s.d.)	24.0 (3.2)	24.8 (4.5)	25.2 (4.3)	26.9 (4.9)	0.006

Hospital Anxiety and Depression Scale, Anxiety: mean (s.d.)	10.1 (3.6)	12.1 (3.7)	13.4 (3.6)	14.0 (3.6)	<0.001

Hospital Anxiety and Depression Scale, Depression: mean (s.d.)	6.7 (3.7)	8.2 (4.1)	10.0 (4.4)	12.4 (4.7)	<0.001

Social Functioning Questionnaire, mean (s.d.)	5.9 (3.4)	8.6 (4.0)	11.3 (4.4)	12.7 (3.8)	<0.001

Total cost (preceding 6 months), mean (s.d.)	2405.2 (2526.3)	2601.8 (2837.2)	2668.1 (2887.1)	2692.5 (2708.3)	0.954

a. Where 0, no personality dysfunction; 1, personality difficuity; 2, mild personality disorder; 3, moderate personality disorder; and 4, severe personality disorder.

b. χ^2^ for gender differences, ANOVA for others.

**Fig. 1:**
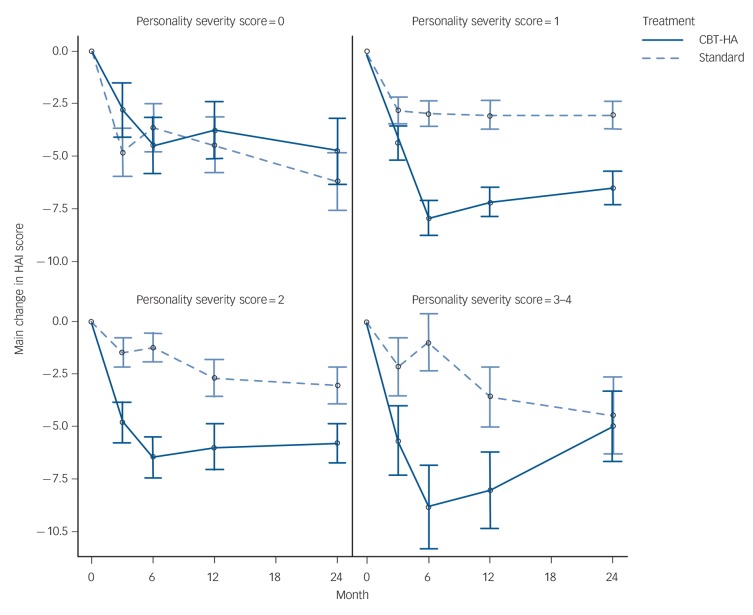
Mean change in scores on the short form of the Health Anxiety Inventory (HAI) separated by personality status. Personality severity scores: 0, no personality dysfunction (*n* = 63); 1, personality difficulty (*n* = 197); 2, mild personality disorder (*n* = 142); 3–4, moderate and severe personality disorder (*n* = 42).

The outcome data over follow-up by ICD-11 classification are detailed in Table 2 and in Fig. 1. Contrary to our hypotheses the results show that those with no personality dysfunction showed no benefit from CBT-HA compared with standard care at any time point in the study; overall, standard care was superior for the symptoms of anxiety and depression (*P*<0.01) and for social functioning (*P*<0.05) but not for health anxiety. For all other groups the picture was different. For participants with personality difficulty and mild personality disorder, there was evidence of much stronger reduction in health anxiety in the CBT-HA group at all time points compared with standard care (*P*<0.001). For participants with moderate and severe personality disorder the initial benefit was not retained at 2 years resulting in a less strong relationship over follow-up (*P*<0.05). Clinical symptomatology increased and social dysfunction was greater with each increment of personality pathology (Table 1). The differences in scores between treatment groups were most marked for health anxiety; similar, but lower, differences were found for generalised anxiety and depressive symptoms (Table 2).

**Table 2:** Summary changes from baseline in the cognitive–behavioural therapy for health anxiety (CBT-HA) group compared with standard care in ICD-11 personality groups^a^

	ICD-11 personality level, difference (95% CI)			
	0 (*n* = 63)	1 (*n* = 197)	2 (*n* = 142)	3 and 4 (*n* = 42)
Health Anxiety Inventory scores				
3 month	2.12 (−1.41 to 5.64)	−1.62 (−3.56 to 0.31)	−3.24** (−5.57 to −0.92)	−3.76 (−8.24 to 0.71)
6 month	−1.29 (−4.9 to 2.31)	−4.80*** (−6.77 to −2.84)	−5.51*** (−7.85 to −3.16)	−8.13** (−12.59 to −3.66)
12 month	0.47 (−3.11 to 4.05)	−3.55** (−5.54 to −1.57)	−3.32** (−5.69 to −0.96)	−4.42 (−8.99 to 0.14)
24 month	1.45 (−2.12 to 5.03)	−2.98** (−4.98 to −0.97)	−2.96* (−5.35 to −0.56)	−0.31 (−4.93 to 4.31)
At all time points	0.69 (−2.23 to 3.6)	−3.24*** (−4.84 to −1.64)	−3.76*** (−5.78 to −1.74)	−4.16* (−7.99 to −0.33)

Hospital Anxiety and Depression Scale, anxiety scores				
6 month	1.45 (−0.5 to 3.39)	−1.47* (−2.64 to −0.29)	−1.71* (−3.05 to −0.36)	−2.03 (−4.7 to 0.65)
12 month	2.68 (0.75 to 4.62)	−1.70** (−2.89 to −0.51)	−1.42*s (−2.78 to −0.06)	−1.32 (−4.05 to 1.41)
24 month	2.21* (0.25 to 4.16)	−1.81** (−3.01 to −0.60)	−1.00 (−2.38 to 0.38)	−1.00 (−3.77 to 1.77)
At all time points	2.11** (0.51 to 3.71)	−1.66*** (−2.62 to −0.70)	−1.38* (−2.56 to −0.20)	−1.45 (−3.79 to 0.89)

Hospital Anxiety and Depression Scale, depression scores				
6 month	2.17* (0.11 to 4.24)	−1.33* (−2.49 to −0.17)	−1.1 (−2.49 to 0.28)	−1.42 (−4.32 to 1.48)
12 month	1.79 (−0.27 to 3.85)	−1.27* (−2.45 to −0.09)	−0.69 (−2.10 to 0.71)	−2.64 (−5.62 to 0.33)
24 month	3.29** (1.22 to 5.36)	−0.69 (−1.88 to 0.49)	−1.84* (−3.27 to −0.41)	−2.06 (−5.08 to 0.96)
At all time points	2.42** (0.62 to 4.21)	−1.10* (−2.06 to −0.13)	−1.21* (−2.41 to −0.02)	−2.04 (−4.55 to 0.47)

Social Functioning Questionnaire scores				
6 month	2.32* (0.19 to 4.45)	−0.42 (−1.57 to 0.73)	−0.31 (−1.68 to 1.06)	−1.89 (−4.54 to 0.77)
12 month	1.52 (−0.61 to 3.64)	−0.08 (−1.25 to 1.09)	−0.66 (−2.05 to 0.73)	−1.91 (−4.64 to 0.82)
24 month	2.88** ( 0.75 to 5.01)	−0.19 (−1.37 to 0.98)	−1.49* (−2.9 to −0.08)	−0.88 (−3.65 to 1.89)
At all time points	2.24* (0.41 to 4.07)	−0.23 (−1.19 to 0.73)	−0.82 (−1.97 to 0.33)	−1.56 (−3.83 to 0.71)

a. All minus scores indicate greater improvement in CBT-HA group except for social functioning (SFQ)). All analyses used a mixed-model approach with time, treatment group and time × treatment interaction as fixed effects, baseline measurement as covariate, and patient as random effect by personality severity group. ICD-11 personality level : 0, no personality dysfunction; 1, personality difficulty; 2, mild personality disorder; 3, moderate personality disorder; and 4, severe personality disorder. Full details of all scores are shown in online Table DS2–4.

* *P*<0.05,

** *P*<0.01,

*** *P*<0.001.

Total costs over 24-month follow-up by randomised group and personality score are detailed in Table 3. Costs were broadly similar across groups, although highest in those with personality dysfunction and lowest in those with moderate to severe personality disorder. Regression analysis showed that the differences in cost between groups fell well short of significance.

**Table 3:** Total costs over 24-month follow-up by randomised group and personality level

ICD-11 personality score	CBT-HA, mean (s.d.)	Treatment as usual, mean (s.d.)	Total, mean (s.d.)	Adjusted mean difference (95% CI)	*P*
No personality disorder, 0 (*n* = 44)	6386.5 (5858.1)	7853.0 (10422.2)	7153.05 (8490.27)	−1232.8 (−6215.66 to 3749.89)	0.620

Personality difficulty, 1 (*n* = 134)	7215.8 (8140.8)	8155.8 (9075.8)	7657.76 (8573.20)	−380.1 (−3167.13 to 2406.93)	0.788

Mild personality disorder, 2 (*n* = 86)	6792.7 (5739.6)	7667.0 (10012.3)	7657.77 (8573.20)	471.8 (−2377.20 to 3300.87)	0.747

Moderate and severe personality disorder, 3+4 (*n* = 23)	6610.2 (3392.4)	5391.8 (3545.6)	6080.48 (3435.36)	1262.4 (−1869.75 to 4394.54)	0.410

CBT-HA, cognitive–behavioural therapy for health anxiety.

## Discussion

### Surprising nature of findings

The challenging finding of this study was that both the hypotheses concerning personality status were soundly contradicted. These results should be seen in the context of the main primary aim of the trial; in the analysis of outcomes independently of personality status CBT-HA was markedly superior to standard care with respect to clinical symptoms of anxiety (and to some extent depression) but no marked changes were found in social function.^36^ People with no personality dysfunction did not benefit from CBT-HA and as their social functioning deteriorated with CBT this treatment cannot be regarded as effective in this population. By contrast those with any form of personality abnormality (personality difficulty, mild or moderate personality disorder) did benefit from CBT-HA and their improvement was maintained over 2 years except in those with moderate or severe personality disorder. Such a finding has not been reported before and as it contradicted our main hypothesis (with the possible exception of worse outcome in more severe personality disorder) other explanations need to be considered before it can be accepted as valid.

In the report from the original trial^36^ the costs were equivalent in both treatment groups and no clear saving was made with CBT-HA. A large part of the costs was taken up with the care of patients with severe medical illness and this may have disguised any savings made by CBT-HA. Nonetheless, the costs were less, but not significantly so, in all groups receiving CBT-HA compared with standard care, with the exception of those with moderate and severe personality disorder who cost more in the CBT-HA group (mean £1166) (Table 3). This suggests the possibility that for those who have moderate or severe personality disorder there is a greater cost with CBT-HA, for reasons that are not completely clear but seem to be independent of medical status, and, as suggested in a previous paper,^37^ may be related to poorer social function.

A qualifying comment is necessary here, as in the trial a large proportion of those screened did not agree to take part in the trial.^36^ It could be argued that the 444 patients seen were not therefore representative of the total population.

### ICD-11 classification of personality disorder

It is worth emphasising that this is the first study to report on the effect of personality status using the new ICD-11 coding and so there are no other studies with which this one can be compared. The small number of patients with no personality dysfunction (*n* = 63) may appear surprising but there are other data suggesting that when personality difficulty is taken into account this subsyndromal group accounts for a large proportion of the total.^38–41^ Patients with health anxiety commonly have symptoms for many years before they present for treatment^41^ and people with chronic anxiety conditions have a high prevalence of personality disorder^42^ and so the overall prevalence of personality disorder of 42% in this sample is in keeping with other figures. It is also important to stress that the diagnosis of core personality disorder is very little different in ICD-11 from ICD-10, but the great flexibility in diagnosis across the lifespan tends to increase prevalence.^40^ Only one other study has examined the effect of personality status on the outcome of health anxiety and hypochondriasis and this is not directly comparable with our data. This was a follow-up of an RCT of paroxetine, placebo, CBT and those who dropped out. In total, 60% were followed up successfully and those who had less harm avoidance and were more cooperative had a better outcome.^43^ No analysis by treatment group was made.

It also could be argued in our study that a proportion of the population may have been misdiagnosed with health anxiety and this could be explained by the cut-off of 20 points on the HAI as being too low. This score equates to around 62 on the long version of the HAI, and a score of 67 on the long HAI has been found to be a good cut-off point for discriminating between severe and less severe health anxiety.^44^ But as all patients satisfied the criteria for hypochondriasis it is unlikely the level of health anxiety was too low. The requirement for high scores on health anxiety and the diagnosis of hypochondriasis would only exclude patients with a primary depression component to their symptoms. Third, it could be argued that the patients with no personality dysfunction had appropriate health anxiety because of incipient and concurrent significant medical illness but this view is not supported by the figures as the costs were lower in those with no personality dysfunction compared with those with personality difficulty and personality disorder, although none of these differences was statistically significant.

It is also fair to add that the ICD-11 classification of personality disorder is not yet approved by the World Health Organization and more is currently being done to confirm the cut-off points for the levels of personality disturbance.^45^ The fundamental structure of the reclassification is nonetheless very likely to be approved. The version of the ICD-11 classification used in the study^28^ shows some slight differences to the current version, but not to any substantive degree. It is also worth pointing out that if the analysis of this study had been carried out using the old ICD-10 diagnostic system of ‘personality disorder’ *v.* ‘no personality disorder’, it is unlikely that these important differences would have been found, as the markedly positive value of CBT-HA in mild personality disorder and personality difficulty would have been split between the groups.

There are also other suggestions that personality status may improve during the course of psychological treatment for health anxiety, and although one needs to be aware of the well-established evidence that standard personality measures tend to improve as mood improves^46^ there are some reasons to think that the change is more substantial.^47^

### Implications for treatment

Taken together, but subject to further replication studies, it is reasonable to suggest that in the absence of personality dysfunction CBT-HA is an inappropriate treatment for health anxiety. It also implies that an assessment of personality status is necessary in the evaluation of people with suspected pathological health anxiety, as those without any personality disturbance may be much more appropriately treated, as at present, with reassurance and support rather than formal psychological intervention. The findings also give some clinical credence to the notion of a subsyndromal form of personality dysfunction in the form of personality difficulty, as the differences in outcome between this group and those with no personality dysfunction were so different.

In contrast with studies in depression and anxiety,^9^ we found little evidence that personality disorder had an impact on service use and cost. The results in terms of costs present a mixed picture, one which reflects those of the main study. Clear conclusions regarding differences in cost are difficult to make in this group because, irrespective of health anxiety and personality status, study participants often had substantial physical health problems that result in substantial levels of service use and therefore high costs. The relative influence of personality on service-use behaviour may therefore be limited. Previously reported evidence of greater service use at all levels of services in patients with personality disorder, especially at levels of greater severity,^38^ is slightly at variance with our findings, which showed lower costs in more severe personality disorder, again illustrating the likely impact of comorbid medical conditions on total cost.

Clinical psychiatry has not yet embraced the importance of twin assessments of mental state and personality status when deciding on treatment but this study, and others across a wide range of conditions^3–8,10,11,48,49^ suggest that better judgements can be made when personality status is given as much importance as mental state in clinical practice.

## Appendix Summary of ICD-11 classification used in coding personality status

**Table:**

Diagnostic status in ICD-11	No personality disorder	Subsyndromal (classified as a Z-code) but not as a personality disorder	Personality disorder		
Dimensional level	Level 0	Level 1	Level 2	Level 3	Level 4
Description	No personality dysfunction	Personality difficulty	Mild personality disorder	Moderate personality disorder	Severe personality disorder

Main defining features	No evidence of any personality dysfunction in any setting	Some problems of personality functioning with distress confined to specific settings only	Clear evidence of general personality dysfunction but social and occupational roles maintained	More severe personality dysfunction with major disruption of relationships and function; some risk of harm to self and/or others	Major personality dysfunction with inability to maintain relationships, and loss of societal roles; high risk of harm to self or others

(for latest status of classification see Tyrer *et al*^45^)

## References

[1] Tyrer P Ferguson B Fowler-Dixon R Kelemen A A plea for the diagnosis of hypochondriacal personality disorder. J Psychosom Res 1990; 34: 637–42. 2290136 10.1016/0022-3999(90)90107-f

[2] Tyrer P Cooper S Crawford M Dupont S Green J Murphy D Prevalence of health anxiety problems in medical clinics. J Psychosom Res 2011; 71: 392–4. 22118381 10.1016/j.jpsychores.2011.07.004

[3] Newton-Howes G Tyrer P Johnson T Personality disorder and the outcome of depression: a meta-analysis of published studies. Br J Psychiatry 2006; 188: 13–20. 16388064 10.1192/bjp.188.1.13

[4] Newton-Howes G Tyrer P Johnson T Mulder R Kool S Dekker J Influence of personality on the outcome of treatment in depression: systematic review and meta-analysis. J Pers Disord 2014; 28: 577–93. 24256103 10.1521/pedi_2013_27_070

[5] Tyrer P Seivewright H Johnson T The Nottingham Study of Neurotic Disorder: predictors of 12 year outcome of dysthymic, panic and generalised anxiety disorder. Psychol Med 2004; 34: 1385–94. 15724870 10.1017/s0033291704002569

[6] Telch MJ Kamphuis JH Schmidt NB The effects of comorbid personality disorders on cognitive behavioral treatment for panic disorder. J Psychiatr Res 2011; 45: 469–74. 20880547 10.1016/j.jpsychires.2010.08.008

[7] van Noorden MS van Fenema EM van der Wee NJ van Rood YR Carlier IV Zitman FG Predicting outcomes of mood, anxiety and somatoform disorders: the Leiden routine outcome monitoring study. J Affect Disord 2012; 142: 122–31. 22840464 10.1016/j.jad.2012.03.051

[8] Thiel N Hertenstein E Nissen C Herbst N Külz AK Voderholzer U The effect of personality disorders on treatment outcomes in patients with obsessive-compulsive disorders. J Pers Disord 2013; 27: 697–715. 23795757 10.1521/pedi_2013_27_104

[9] Knerer G Byford S Johnson T Seivewright H Tyrer P The Nottingham study of neurotic disorder: predictors of 12 year costs. Acta Psychiatr Scand 2005; 112: 224–32. 16095478 10.1111/j.1600-0447.2005.00552.x

[10] Tyrer P Seivewright N Ferguson B Murphy S Johnson AL The Nottingham study of neurotic disorder. Effect of personality status on response to drug treatment, cognitive therapy and self-help over two years. Br J Psychiatry 1993; 162: 219–26. 8435693 10.1192/bjp.162.2.219

[11] Fournier JC DeRubeis RJ Shelton RC Gallop R Amsterdam JD Hollon SD Antidepressant medications *v.* cognitive therapy in people with depression with or without personality disorder. Br J Psychiatry 2008; 192: 124–9. 18245030 10.1192/bjp.bp.107.037234 PMC2682552

[12] Tyrer P Seivewright N Seivewright H Long term outcome of hypochondriacal personality disorder. J Psychosom Res 1999; 46: 177–85. 10098826 10.1016/s0022-3999(98)00072-5

[13] Starcevic V Hypochondriasis and health anxiety: conceptual challenges. Br J Psychiatry 2013; 202: 7–8. 23284146 10.1192/bjp.bp.112.115402

[14] Fergus DA Valentiner DP Disease phobia and disease conviction are separate dimensions underlying hypochondriasis. J Behav Ther Exp Psychiatry 2010; 41: 438–44. 20627267 10.1016/j.jbtep.2010.05.002

[15] Tyrer P Tyrer H The departure of hypochondriasis is no loss. Aust NZ J Psychiatry 2014; 48: 772–3. 10.1177/0004867414540755 25048652

[16] American Psychiatric Association Illness anxiety. In Diagnostic and Statistical Manual of Mental Disorders (5th edn) (DSM-5): 315 APA, 2013.

[17] Clark DM Salkovskis PM Hackman A Wells A Fennell M Ludgate J Two psychological treatments for hypochondriasis: randomised controlled trial. Br J Psychiatry 1998; 173: 218–25. 9926097 10.1192/bjp.173.3.218

[18] Fallon BA Harper KM Landa A Pavlicova M Schneier FR Carson A Personality disorders in hypochondriasis: prevalence and comparison with two anxiety disorders. Psychosomatics 2012; 53: 566–74. 22658329 10.1016/j.psym.2012.02.002 PMC3449016

[19] Tyrer P Cooper S Tyrer H Salkovskis P Crawford M Green J CHAMP: cognitive behaviour therapy for health anxiety in medical patients: a randomised controlled trial. BMC Psychiatry 2011; 11: 99. 21672205 10.1186/1471-244X-11-99 PMC3141642

[20] Salkovskis PM Rimes KA Warwick HMC Clark DM The Health Anxiety Inventory: development and validation of scales for the measurement of health anxiety and hypochondriasis. Psychol Med 2002; 32: 843–53. 12171378 10.1017/s0033291702005822

[21] Seivewright H Prevalence and Treatment of Health Anxiety in Genitourinary Medicine. PhD thesis: Imperial College London, 2009.

[22] First MB Spitzer RL Gibbon M Williams JB Structured Clinical Interview for the DSM-IV Axis I Disorders. American Psychiatric Press, 1996.

[23] Zigmond AS Snaith RP The Hospital Anxiety and Depression Scale. Acta Psychiatr Scand 1983; 57: 361–70. 6880820 10.1111/j.1600-0447.1983.tb09716.x

[24] EuroQol Group EuroQol – a new facility for the measurement of health-related quality of life. Health Policy 1990; 16: 199–208. 10109801 10.1016/0168-8510(90)90421-9

[25] Tyrer P Nur U Crawford M Karlsen S McLean C Rao B The Social Functioning Questionnaire: a rapid and robust measure of perceived functioning. Int J Soc Psychiatr 2005; 51: 265–75. 16252794

[26] Barrett B Byford S Crawford M Patton R Drummond C Henry JA Cost-effectiveness of referral to an alcohol health worker in patients attending an accident and emergency department: a decision-making approach. Drug Alcohol Depend 2006; 81: 47–54. 16006055 10.1016/j.drugalcdep.2005.05.015

[27] Tyrer P Quick Personality Assessment Schedule: PAS-Q. In Personality Disorders: Diagnosis, Management and Course (2nd edn) (ed Tyrer P): 181–90. Arnold, 2000.

[28] Tyrer P Crawford M Mulder R Blashfield R Farnam A Fossati A The rationale for the reclassification of personality disorder in the 11th Revision of the International Classification of Diseases. Personal Ment Health 2011; 5: 246–59.

[29] Tyrer P Coombs N Ibrahimi F Mathilakath A Bajaj P Ranger M Critical developments in the assessment of personality disorder. Br J Psychiatry 2007; 190 (suppl 49): s51–9. 10.1192/bjp.190.5.s51 17470943

[30] Curtis L Unit Costs of Health and Social Care, 2010. PSSRU, University of Kent, 2011.

[31] British Medical Association & Royal Pharmaceutical Society of Great Britain BNF 59. BMJ Books/Pharmaceutical Press, 2010.

[32] Department of Health NHS Reference Costs. London: Department of Health, 2011.

[33] National Institute for Health and Care Excellence Guide to the Methods of Technology Appraisal. NICE, 2008. 27905712

[34] Barber JA Thompson SG Analysis and interpretation of cost data in randomised controlled trials: review of published studies. BMJ 1998; 317: 1195–200. 9794854 10.1136/bmj.317.7167.1195 PMC28702

[35] Efron B Tibshirani R An Introduction to the Bootstrap. Chapman and Hall, 1993.

[36] Tyrer P Cooper S Salkovskis P Tyrer H Crawford M Byford S Clinical and cost-effectiveness of cognitive behaviour therapy for health anxiety in medical patients: a multicentre randomised controlled trial. Lancet 2014; 383: 219–25. 24139977 10.1016/S0140-6736(13)61905-4

[37] Barrett B Tyrer P Tyrer H Cooper S Crawford MJ Byford S An examination of the factors that influence costs in medical patients with health anxiety. J Psychosom Res 2012; 73: 59–62. 22691561 10.1016/j.jpsychores.2012.04.014

[38] Yang M Coid J Tyrer P Personality pathology recorded by severity: national survey. Br J Psychiatry 2010; 197: 193–9. 20807963 10.1192/bjp.bp.110.078956

[39] Kim YR Blashfield R Tyrer P Hwang ST Lee HS Field trial of a putative research algorithm for diagnosing ICD-11 personality disorders in psychiatric patients: 1. Severity of personality disturbance. Personal Ment Health 2014; 8: 67–78. 24532556 10.1002/pmh.1248

[40] Tyrer P Crawford M Sanatinia R Tyrer H Cooper S Muller-Pollard C Preliminary studies of the ICD 11 classification of personality disorder in practice. Personal Ment Health 2014; 8: 254–63. 25200623 10.1002/pmh.1275

[41] Hedman E Andersson G Andersson E Ljótsson B Rück C Asmundson GJG Internet-based cognitive–behavioural therapy for severe health anxiety: randomised controlled trial. Br J Psychiatry 2011; 198: 230–6. 21357882 10.1192/bjp.bp.110.086843

[42] Latas M Milovanovic S Personality disorders and anxiety disorders: what is the relationship? Curr Opin Psychiatry 2014; 27: 57–61. 24270478 10.1097/YCO.0000000000000025

[43] Greeven A van Balkom AJ Spinhoven P Personality predicts time to remission and clinical status in hypochondriasis during a 6-year follow-up. J Nerv Ment Dis 2014; 202: 402–7. 24727716 10.1097/NMD.0000000000000133

[44] Hedman E Lekander M Ljótsson B Lindefors N Rück C Andersson G Optimal cut-off points on the Health Anxiety Inventory, Illness Attitude Scales and Whiteley Index to identify severe health anxiety. PLoS One 2015; 10: e0123412. 25849477 10.1371/journal.pone.0123412 PMC4388630

[45] Tyrer P Reed GM Crawford M Classification, assessment, prevalence, and effect of personality disorder. Lancet 2015; 385: 717–26. 25706217 10.1016/S0140-6736(14)61995-4

[46] Coppen AL Metcalfe H The effect of a depressive illness on MPI scores. Br J Psychiatry 1965; 111: 236–9. 14288070 10.1192/bjp.111.472.236

[47] Hedman E Andersson G Lindefors N Gustavsson P Lekander M Rück C Personality change following internet-based cognitive behavior therapy for severe health anxiety. PLoS One 2014; 9: e113871. 25437150 10.1371/journal.pone.0113871 PMC4250052

[48] Tyrer P Yang M The clinical implications of personality-generated mental illness. Personal Ment Health 2015; 9: 17–20. 25711647 10.1002/pmh.1286

[49] Koelen JA Luyten P Eurelings-Bontekoe LH Diguer L Vermote R Lowyck B The impact of level of personality organization on treatment response: a systematic review. Psychiatry 2012; 75: 355–74. 23244013 10.1521/psyc.2012.75.4.355

